# Performance of Polyester-Based Electrospun Scaffolds under In Vitro Hydrolytic Conditions: From Short-Term to Long-Term Applications

**DOI:** 10.3390/nano9050786

**Published:** 2019-05-22

**Authors:** Oscar Gil-Castell, José David Badia, Jordi Bou, Amparo Ribes-Greus

**Affiliations:** 1 Instituto de Tecnología de Materiales (ITM), Universitat Politècnica de València, Camino de Vera s/n, 46022 Valencia, Spain; ogilcastell@doctor.upv.es (O.G.-C.); jose.badia@uv.es (J.D.B.); 2 Departament d’Enginyeria Química, Escola Tècnica Superior d’Enginyeria, Universitat de València, Av. de la Universitat s/n, 46100 Burjassot, Spain; 3 Departament d’Enginyeria Química, Universitat Politècnica de Catalunya, Av. Diagonal 647 (ETSEIB), 08028 Barcelona, Spain; Jordi.bou@upc.edu

**Keywords:** biopolymer, polyester, PLGA, PCL, PDO, PHB, scaffolds, tissue engineering, in vitro hydrolytic degradation

## Abstract

The evaluation of the performance of polyesters under in vitro physiologic conditions is essential to design scaffolds with an adequate lifespan for a given application. In this line, the degradation-durability patterns of poly(lactide-co-glycolide) (PLGA), polydioxanone (PDO), polycaprolactone (PCL) and polyhydroxybutyrate (PHB) scaffolds were monitored and compared giving, as a result, a basis for the specific design of scaffolds from short-term to long-term applications. For this purpose, they were immersed in ultra-pure water and phosphate buffer solution (PBS) at 37 °C. The scaffolds for short-time applications were PLGA and PDO, in which the molar mass diminished down to 20% in a 20–30 days lifespan. While PDO developed crystallinity that prevented the geometry of the fibres, those of PLGA coalesced and collapsed. The scaffolds for long-term applications were PCL and PHB, in which the molar mass followed a progressive decrease, reaching values of 10% for PCL and almost 50% for PHB after 650 days of immersion. This resistant pattern was mainly ascribed to the stability of the crystalline domains of the fibres, in which the diameters remained almost unaffected. From the perspective of an adequate balance between the durability and degradation, this study may serve technologists as a reference point to design polyester-based scaffolds for biomedical applications.

## 1. Introduction

The polyesters are some of the most used polymeric materials for biomedical applications such as sutures, implants, artificial skin and controlled drug release [1,2,3,4,5]. Such is the case of the use of polylactide (PLA), polyglycolide (PGA), polycaprolactone (PCL), poly(lactide-*co*-glycolide) (PLGA), polydioxanone (PDO) or polyhydroxybutyrate (PHB). These polymeric materials degrade by hydrolytic processes and result in low molar mass species, that can be bioabsorbed and metabolised by the human body.

The irruption of electrospinning in tissue engineering has boosted the technology of production of biomaterials based on architectures of fibres, in which diameters can vary from the micrometric scale to hundreds of nanometres [6,7,8], mimicking the native extracellular matrix and allowing enough porosity to facilitate cellular growth [9]. Precisely, these scaffolds are required to ensure an appropriate balance between enough time of structural endurance to permit angiogenesis, and suitable degradation profiles to be decomposed avoiding an inflammatory response and eluding the delivery of toxic low molar mass compounds. These degradation by-products may affect healthy cells as well as interact with sensitive substances such as proteins and peptides or drugs [10].

Monitoring and understanding the physico-chemical processes underwent by biopolymeric scaffolds along their exposure to physiological conditions is essential to ensure their performance during application. In this sense, the study of their degradation profiles is necessary to guarantee the desired behaviour, according to the biomedical purpose, such as skin reparation [11,12] or cardiac surgeries [13,14,15], among others, which will need different times of endurance [16]. For instance, dissimilar regeneration periods may be considered according to the renewal rate on different tissues of the human body: 2–9 days for stomach cells, 10–30 days for skin epidermis cells, 30–60 days for trachea cells or 180 days for bone osteoblasts [17].

In the balance of the performance-to-degradation ratio of the scaffolds, the polymer composition plays an important role. Polymers from different origins and physico-chemical features have been proposed for tissue engineering [17]. Among them, natural polymers produced by microbes such as the polyhydroxybutyrate (PHB), as well as synthetic polymers such as the poly(lactide-co-glycolide) (PLGA), the polycaprolactone (PCL) or the polydioxanone (PDO) have been widely used for scaffold development. All of them are known to be resorbable, absorbable or bioabsorbable when implanted in the living body. However, their differences in the crystalline morphology may determine its hydrolytic degradation behaviour.

In vitro experiments are relevant to test and compare the performance of biopolymeric materials and assess their durability [18,19]. When the aqueous solution penetrates into the polymer, it swells incrementing the dimensions of the interface of contact, which promotes further degradation. The mechanism of degradation of polyesters under abiotic aqueous environments takes place through hydrolysis of the ester bonds, auto-catalysed by carboxylic groups, exponentially increasing along the exposure time [20]. These microstructural changes induce the formation of macroscopic pores or cracks, the loss of monomeric and oligomeric species, thus reducing the mass of the polymers and finally decomposing their architecture until bioassimilation or excretion [21]. Accordingly, after a successful growing and spreading of the cells into the scaffold, polyester-based scaffolds are expected to degrade, disintegrate and be assimilated by the patient.

Characterisation studies of the in vitro degradation of the most used polyesters for biomedical applications in bulk have been reported, with respect to its water-uptake rate, degradation kinetics and mechanisms, morphology and physico-chemical properties [22]. As electrospun scaffolds, some characterisation studies of in vitro degradation generally consider the monitoring of mass-loss and molar-mass along with hydrolytic exposure. The in vitro behaviour under physiological conditions for PLGA [14,23,24], PDO [25], PCL [26] and PHB [27] scaffolds has been individually assessed. These studies are usually carried out in the first stages of immersion and complete degradation or disintegration is not commonly addressed. Therefore, a comparison of the disintegration behaviour of these polymers under identic simulated conditions may offer a broader vision of their applicability, having in mind the expected lifetime for the future specific application [10].

The aim of this study was, therefore, to compare the in vitro degradation behaviour of PLGA, PCL, PDO and PHB scaffolds from an experimental and comprehensive perspective, under simulated physiologic conditions until complete disintegration. For this purpose, electrospun scaffolds were subjected to ultra-pure water and phosphate buffer solution (PBS) at 37 °C. Accordingly, a set of analytical techniques such as pH measurement, gravimetry, size-exclusion chromatography (SEC), differential scanning calorimetry (DSC) and field-emission scanning electron microscopy (FE-SEM) was proposed to point out the appropriate indicators to monitor and compare the in vitro hydrolytic degradation profiles of these polyester-based scaffolds for biomedical purposes.

## 2. Materials and Methods 

### 2.1. Materials

PLGA and PDO were provided by Sigma-Aldrich (Madrid, Spain) under the grades Resomer RG 505 and Resomer X, respectively; PCL was provided by Perstorp (Malmö, Sweden) under the grade CAPA 6800; PHB grade P209 was provided by Biomer (Krailling, Deutschland). Dimethylformamide (DMF), chloroform and hexafluoroisopropanol (HFIP) were used as solvents for electrospinning. For the hydrolytic degradation, ultra-pure water of type 1 (ISO 3696) [28] and Dulbecco’s phosphate buffered saline solution (PBS, D1408) and NaOH 1 M for adjusting pH in PBS, were used. All these reagents, except water, were supplied by Sigma-Aldrich and were used without further purification.

### 2.2. Scaffold Preparation

The fibrous scaffolds were obtained by electrospinning by means of an Yflow Electrospinner 2.2.D-350 (Málaga, Spain). It consisted of double polarization, integrated drum collector, control panel and robotized stage to move the electrospinning source in an alternative fashion covering a 400 × 400 mm^2^ area. The solutions were prepared at different conditions, as described in Table 1. The solution jet emerging from the stainless steel wire used as the positive electrode (0.9 mm inner diameter) was collected on a waxed paper. Scaffolds were dried over 12 h under vacuum to facilitate the removal of residual organic solvents and moisture and were stored for further analyses.

### 2.3. In Vitro Degradation Methodology

All the electrospun scaffolds were subjected to hydrolytic degradation, by exposition to ultra-pure water and phosphate buffer saline solution (PBS), according to the international norm ISO 10993-13:2010, method 4.3 [29]. Shortly, the initial electrospun scaffolds were cut into rectangular specimens with a mass around 10 mg. The specimens were weighed (*m_0_*) and placed in a previously weighed vial (*m_vial_*). Ten millilitre of degradation medium were introduced and then the vials were sealed with polytetrafluoroethylene (PTFE) threaded plugs and placed in a thermostatically controlled oven at 37 °C. The pH of the PBS solution was adjusted to 7.4 with NaOH 1 M. Between twelve and fifteen extractions were considered along the hydrolytic degradation for each scaffold up to disintegration. Due to the different disintegration timespan of the studied scaffolds, two different time scales were considered, designated as short-term and long-term behaviour.

In order to monitor the process, after certain periods, the scaffolds were withdrawn from the environment by triplicates. The solid and liquid fractions were separated. The specimens coming from the saline buffer were washed with deionised water and then, along with those from water environment, were dried under vacuum to constant mass into the vials (*m_dry_*) and saved for further analyses. The residual mass of the samples (*% mass*) was determined by Equation (1),

(1) $$\%\;mass=\frac{m_{dry}-m_{vial}}{m_{0}}\times 100$$

where *m*_0_, *m_vial_* are the initial mass of the specimen and the vial, respectively, and *m_dry_* is the mass of the vacuum dry assembly sample-vial after degradation.

The pH of the degradation media was measured at room temperature by means of a Crison pH 25 device. Three buffer solutions from Crison were used to calibrate pH-meter: pH 4.01 (phthalate buffer solution), pH 7.00 (phosphate buffer solution), pH 10.01 (borate buffer solution).

### 2.4. Scaffold Characterisation

#### 2.4.1. Size Exclusion Chromatography (SEC)

Size exclusion chromatography (SEC) analyses were carried out by means of an Agilent Infinity 1260 chromatograph (Basel, Switzerland). Separation was performed with a Jordi Associates mixed bed fluorinated column (permeation range: 100–10 × 10^6^ Dalton). All samples were dissolved in mobile phase containing 2.72 g·L^−1^ of sodium trifluoroacetate (NaTFA). This solvent was previously degassed by vacuum filtration over PTFE 0.45 µm pore membranes. The flow rate was set at 1 mL·min^−1^. One hundred microlitre samples of about 0.1% concentration were injected. Detection was conducted by UV-vis-detector. Monodisperse PMMA samples from Sigma Aldrich and Agilent were used for the previous calibration.

#### 2.4.2. Differential Scanning Calorimetry (DSC)

The calorimetric data were obtained by means of a Mettler-Toledo DSC 820^e^ differential scanning calorimeter (Columbus, OH, USA), previously calibrated following the procedure of In and Zn standards. The samples, with a mass of about 4 mg were analysed between 0 and 200 °C with a heating/cooling/heating rate of 10 °C·min^−1^. All the experiments were run under a nitrogen atmosphere with a flow rate of 50 mL·min^−1^. The specimens were characterised at least in triplicate and the averages of temperatures and enthalpies were taken as representative values. The crystallinity degree (*X_c_*) was evaluated from the melting enthalpy results, by means of Equation (2),

(2) $$X_{c}\;\left(\%\right)=\frac{Ζh_{m}}{Ζh_{m}^{0}}\times 100$$

where *∆h_m_* is the melting enthalpy of the sample and *∆h_m_*^0^ is the melting enthalpy of a perfect crystal of the given polymer (PDO 141 J·g^−1^ [30], PCL 148 J·g^−1^ [31] and PHB 146 J·g^−1^ [32]).

#### 2.4.3. Field Emission Scanning Electron Microscopy (FE-SEM)

The surface morphology of the scaffolds was analysed by means of a Zeiss Ultra 55 field emission scanning electron microscope (Oberkochen, Germany). The samples were cut into small pieces and dried at 30 °C in a vacuum oven for 24 h and then kept in a desiccator during 48 h. Afterwards, the specimens were mounted on metal studs and sputter-coated with a platinum layer during 10 s using a Leica EM MED020. The testing was performed at room temperature with a 2 kV voltage. The fibre diameters were measured from the FE-SEM micrographs at random locations (*n = 100*) with the aid of the ImageJ software (National Institutes of Health, Bethesda, MD, USA).

## 3. Results

### 3.1. Initial Properties and Morphology of the Scaffolds

The in vitro degradation behaviour is mainly controlled by the polymer chemistry and physical features of the scaffold. Accordingly, some of the most influent parameters of a scaffold that will determine its subsequent biomedical application were assessed [33,34,35]. The initial surface morphology, fibre diameter, bulk density (ρ) and surface density (ρ_s_), along with the constitutional repeating unit (CRU) are gathered in Table 2.

As the surface morphology revealed, all the scaffolds showed a uniform non-woven fibrous structure. Fibre diameters moved from the nanometric scale for the PLGA to the low micrometric scale of PCL, PDO and PHB. In addition, the diameter distributions of the PLGA and the PDO showed a narrow pattern, while those of the PCL and PHB tended to be wider. Similar bulk and surface densities were observed for all the scaffolds.

### 3.2. Hydrolytic in Vitro Degradation

#### 3.2.1. On-line Monitoring: Changes in the Media 

**Figure 1:**
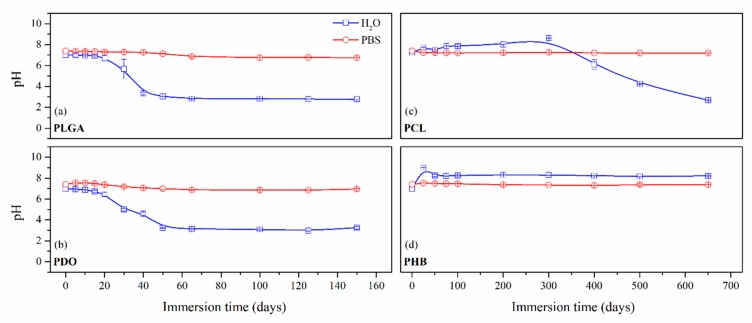
Average pH as a function of the degradation time in ultra-pure water and PBS for PLGA (**a**), PDO (**b**), PCL (**c**) and PHB (**d**) scaffolds.

In order to preliminary assess the hydrolytic degradation of the scaffolds, the evolution of pH was monitored both when immersed in water and PBS. Due to the dissimilar degradation timespan of the studied scaffolds, the results were represented in two different time scales—short-term and long-term degradation—as plotted in Figure 1. According to the experimental design, the generated acidic compounds were measured as accumulation as a function of time. It must be taken into account that the evaluation of the degradation in water may bring information about the hydrolytic mechanism, while the consideration of PBS would better simulate the physiologic conditions.

Concerning the short-term scaffolds, the pH of the PLGA and PDO solutions remained constant during the first stage of immersion. When immersed in water, it dramatically decreased from neutral to acid pH. This drop could be related to a large release of low molar mass acidic monomeric and oligomeric species such as glycolic acid, lactic acid for PLGA and low molar mass hydroxy-acid species for PDO [10,36]. As expected, when immersed into PBS, the pH slightly varied along the immersion due to the intrinsic ability to buffer acidic species released during degradation. Nonetheless, a slight diminution was observed from neutral pH 7.4 to pH 6.6.

About the long-term scaffolds, two different behaviours were found. For PCL scaffolds immersed in water, the pH remained constant along the immersion until day 400, when it started to decrease due to the hypothetic release of acidic species based on hexanoic acid. Indeed, a progressive reduction was observed until the end of the assay, when pH value of 2.7 was found. Conversely, when immersed into PBS, the pH was maintained along the whole assay around 7.4 [37,38]. The hydrolytic degradation of the PHB scaffolds could not be perceived from the evaluation of the pH of the solution media. The pH remained constant in both water and PBS. Although the degradation of the PHB polymer chains ultimately results in 3-hydroxybutyric acid, which can be assimilated by the human organism [39], the slow degradation rate of this material seemed to have prevented the production of acidic low molar mass compounds that would have modified the pH of the media [40].

#### 3.2.2. Impact on the Scaffolds Mass and Molar Mass

**Figure 2:**
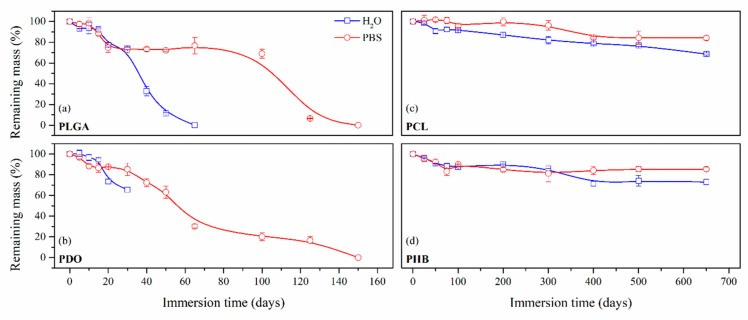
Remaining mass as a function of the degradation time in ultra-pure water and PBS for PLGA (**a**), PDO (**b**), PCL (**c**) and PHB (**d**) scaffolds.

As perceived in the previous section, due to the different degradation performance of the studied scaffolds, the mass evolution along immersion was represented in short-term—PLGA and PDO—and long-term—PCL and PHB—scales, as shown in Figure 2.

On the one hand, the PLGA and PDO scaffolds were completely disintegrated in a relatively short period until day 150. In contrast, the degradation of the PCL and PHB was much slower and took place over a longer period. Even though the scaffolds were not completely dissolved, the monitoring of the degradation was studied until disintegration, which took place around day 650 of immersion both for PCL and PHB scaffolds. After this period, the remnant scaffolds were impossible to handle and the assay was considered as concluded.

**Figure 3:**
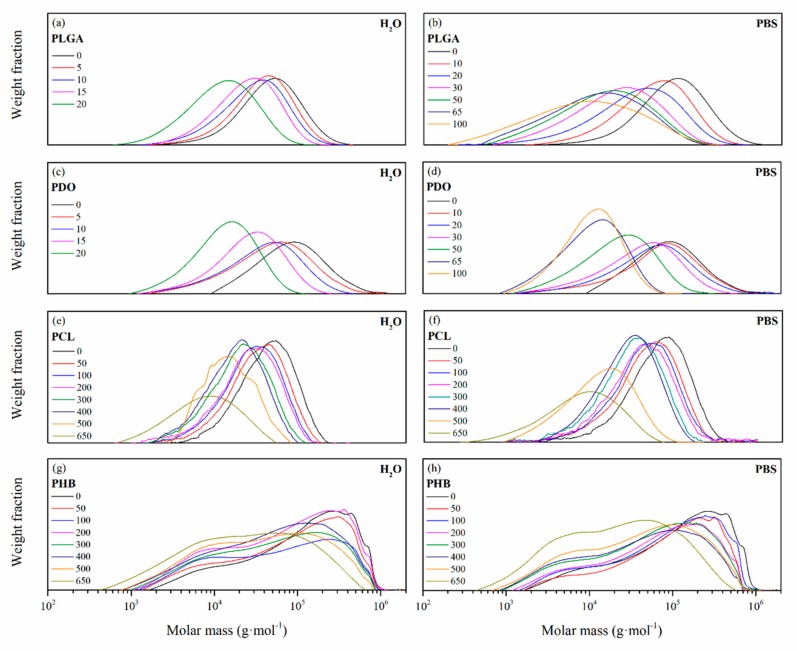
Molar mass distributions as a function of the degradation time in ultra-pure water and PBS for PLGA (**a**), (**b**), PDO (**c**), (**d**), PCL (**e**), (**f**), and PHB (**g**), (**h**) scaffolds.

The degradation behaviour was deeply assessed by means of the variation of the molar mass, which was monitored along the immersion by means of size exclusion chromatography (SEC). The molar mass distributions for the PLGA, PDO, PCL and PHB scaffolds are plotted in Figure 3 as a function of the immersion time. Unimodal distributions were found for PLGA, PDO and PCL scaffolds, while PHB showed a complex multi-modal broad molar mass distribution. It is important to note that samples coming from the hydrolytic degradation with a mass lower than 20% of the initial value were so deteriorated that could not be handled and, subsequently, molar mass analyses were not performed.

In general, the molar mass distributions were displaced towards lower values as a function of the immersion time due to the hydrolytic degradation of the ester bonds. For PLGA and PCL scaffolds, the distributions increased in width along the degradation, turning into a more polydisperse polymer. Random ester bond breakage seemed to take place in all polymeric chains, producing a broader distribution of chain sizes. For the case of the PDO scaffolds, the molar mass distributions reduced their width, turning into a more concise and sharp curve, which can be correlated with a less polydisperse polymer. Some authors found experimental evidence that suggested a preferential degradation of higher molar mass PDO chains, such as tie molecules, that resulted in a more uniform distribution of shorter polymer segments [21,41,42,43]. The broad multi-modal distribution pattern found for PHB showed two main peaks, correlated to low molar mass and high molar mass populations. This behaviour can be associated with the synthetic route of the PHB, in which microorganisms may have produced dissimilar size polymer chains [44,45]. Due to hydrolytic degradation, the PHB scaffolds reduced the high molar mass peak and increased that of low molar mass polymer chains as a function of the immersion time.

**Figure 4:**
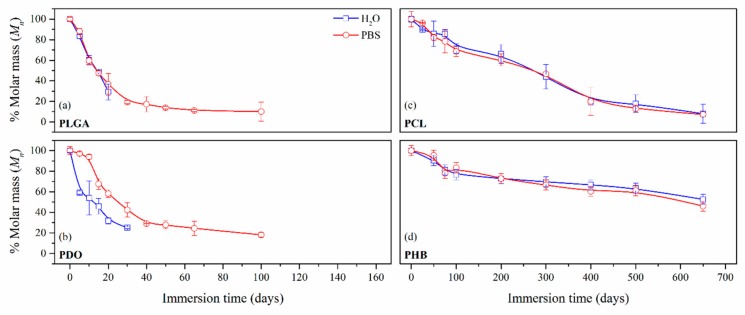
Average molar mass in number (M_n_) percentage as a function of the degradation time in ultra-pure water and PBS for PLGA (**a**), PDO (**b**), PCL (**c**) and PHB (**d**) scaffolds.

The average molar mass in number (*M_n_*) was considered as an appropriate indicator for the comparison of the hydrolytic breakage of the polymer chains along the immersion, which values are gathered in Table 3. However, given the heterogeneity in the molar mass distributions for the different materials, the *M_n_* was normalised, i.e. relative molar mass-loss was calculated, and its evolution as a function of the immersion time was plotted in Figure 4 for a proper comparison.

The decrease of the *M_n_* for the short-term PLGA and PDO scaffolds followed a similar exponential decreasing pattern regardless of the degradation medium, as observed by other authors [36,46,47]. Although the degradation of the PLGA scaffolds in water and PBS followed an identical pattern, for the case of PDO scaffolds a slightly delayed behaviour was found when immersed in PBS. If results are compared to mass loss measurements, one can see that after the *M_n_* reached values near to the 20% of the initial values, a plateau in the remaining mass was found prior to the complete disintegration of both PLGA and PDO scaffolds.

In contrast, long-term PCL and PHB scaffolds showed a similar progressive decrease of the *M_n_* as a function of time. Although the measurements of mass for PCL showed a reduction between 10% and 20% after 650 days of immersion, the molar mass results revealed a massive reduction of *M_n_* of around 80%. However, in PHB scaffolds, the *M_n_* decreased 50% during the 650 days of immersion in both water and PBS. These results might be explained in terms of crystallinity, which might block the diffusion of the hydrolytic media into the polymers and retard the degradation [48]. This hypothesis was confirmed, as latterly shown by the results of differential scanning calorimetry.

#### 3.2.3. Impact on the Scaffolds Crystallinity

**Figure 5:**
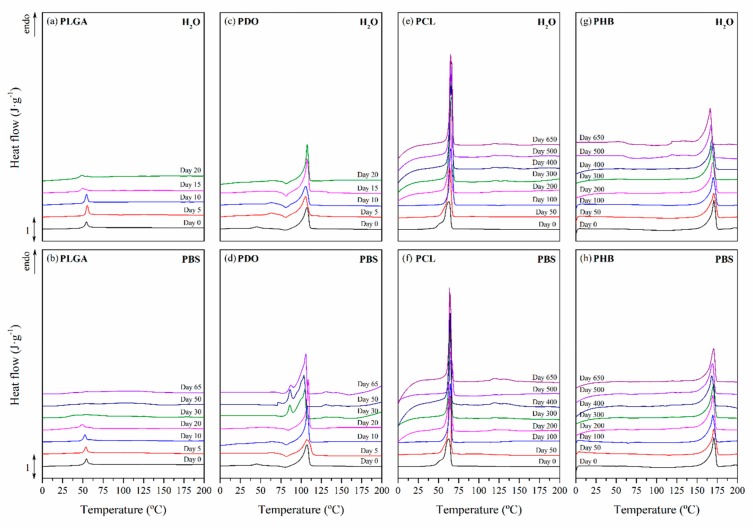
Differential scanning calorimetry traces as a function of immersion time in ultra-pure water and PBS for PLGA (**a**), (**b**), PDO (**c**), (**d**), PCL (**e**), (**f**), and PHB (**g**), (**h**) scaffolds.

The study of the thermal properties is essential to understand the behaviour of polymers subjected to different degrading conditions [49,50,51,52,53,54,55,56,57,58]. The thermal properties of the scaffolds were assessed by means of differential scanning calorimetry (DSC). Figure 5 shows the calorimetric curves of the first heating scan of non-exposed scaffolds and those subjected to in vitro hydrothermal degradation in both media, ultra-pure water and PBS. In order to assure comparison between the different materials, the thermal properties were evaluated from 0 to 200 °C.

**Figure 6:**
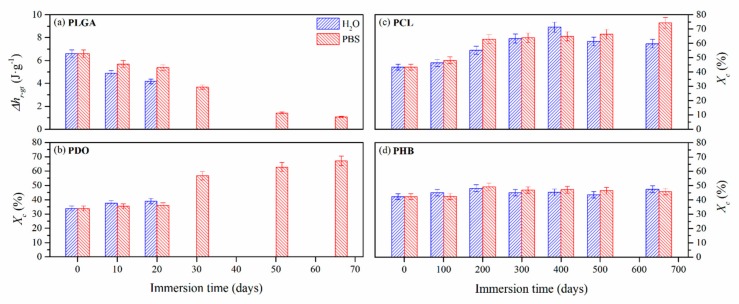
Evolution of the structural relaxation enthalpy (∆h_r-gt_) for PLGA (**a**) and crystallinity degree (X_c_) for PDO (**b**), PCL (**c**) and PHB (**d**) scaffolds as a function of immersion time in ultra-pure water and PBS.

PLGA showed an amorphous morphology, while PDO, PCL and PHB showed a semicrystalline behaviour, regardless of the time subjected to hydrolytic degradation. Due to the different morphology of the selected materials, the endothermic specific enthalpy related to the release of energy during the glass transition (*∆h_r-gt_*) was chosen for PLGA and the crystallinity degree (*X_c_*) for PDO, PCL and PHB scaffolds. The results are plotted in Figure 6. Moreover, Table 4 gathers the peak temperature of the heat release during the glass transition (*T_r-gt_*) for the PLGA and the peak melting temperature (*T_m_*) for the PDO, PCL and PHB scaffolds, closely related to the lamellar thickness (*l_c_*) [59,60].

For the case of the amorphous PLGA scaffolds, a similar but delayed behaviour was found for the endothermic peak associated with the glass transition during immersion in water and PBS. This transition sharpened at short degradation times but reduced its associated enthalpy (*∆h_r-gt_*). Then, it became broader the longer the hydrolytic exposure was until it almost disappeared. As can be seen in Figure 5, particularly when immersed in PBS, the unimodal performance turned into a bimodal transition pattern, which was ascribed to the glass transition of different segments of the copolymer, i.e., polyglycolide (PGA), between 35 and 40 °C, and polylactide (PLA), between 55 and 60 °C [61]. These results are in line with other reports that suggested a preferential hydrolytic breakage of the glycolic-glycolic and glycolic-lactic ester linkages, instead of that of the lactic-lactic bond in random poly(lactide-co-glycolide) [54].

In the same time-scale, PDO scaffolds showed complex semicrystalline behaviour. A cold-crystallisation exothermic peak was perceived immediately before the melting transition. According to Sabino et al., the cold-crystallisation around 80 °C can be ascribed to a partial melting and recrystallization of non-stable crystalline structure [43]. Then, a unimodal melting transition around 108 °C was observed. When immersed in water, the cold-crystallisation remained constant and the melting enthalpy slightly increased, suggesting that crystallinity was developed during immersion [62]. The melting temperature (*T_m_*) slightly decreased, ascribed to a more imperfect crystalline structure with lower lamellar thickness. However, when immersed in PBS, the cold-crystallisation peak disappeared as a function of time and the melting peak turned into a bimodal pattern, suggesting the existence of dissimilar crystalline populations. Highly degraded PDO segments with lower entanglement capability, seemed to have rearranged into an imperfect crystalline structure that melted at lower temperatures, around 86 °C [22]. The global crystallinity degree increased along the immersion from 35% up to 60%.

The PCL scaffolds showed a typical semi-crystalline behaviour with a melting transition around 63 °C. No significant differences were perceived when immersed in water and PBS. In both media, the melting peak sharpened and increased the associated crystallinity degree, from 40% up to 70% after 650 days of immersion. Shorter hydrolysed molecular PCL segments rearranged and crystallised as a function of time [33]. Given the small increase in the melting temperature, slightly more perfect crystals with higher lamellar thickness were developed during immersion [63].

The evaluation of the thermal properties of the PHB scaffolds revealed a semicrystalline behaviour with a melting peak around 171 °C, which was slightly displaced towards lower temperatures, correlated to more imperfect crystals as a function of the immersion time, due to the progress of the hydrolytic degradation. However, the crystallinity degree slightly increased from 42% to 47% after 650 days of immersion in both media [64]. These results suggested that the microstructure of the PHB scaffolds remained almost stable during the immersion period considered in this study.

The hydrolytic process of semicrystalline polyesters occurred in two stages: (i) the attack of the less compacted amorphous regions where diffusion of the hydrolytic medium is easier and faster and (ii) the attack on the crystalline regions, usually hydrophobic and impermeable to the diffusion and penetration of the aqueous media [33,34]. The crystalline fraction of the long-term scaffolds may have been relevant in terms of the increase in the difficulty of the hydrolytic media to penetrate, swell and scissor the polymeric chains. It was therefore in agreement with the resilience of the *M_n_* and mass of the PCL and PHB scaffolds.

#### 3.2.4. Impact on the Scaffolds Surface Morphology

**Figure 7:**
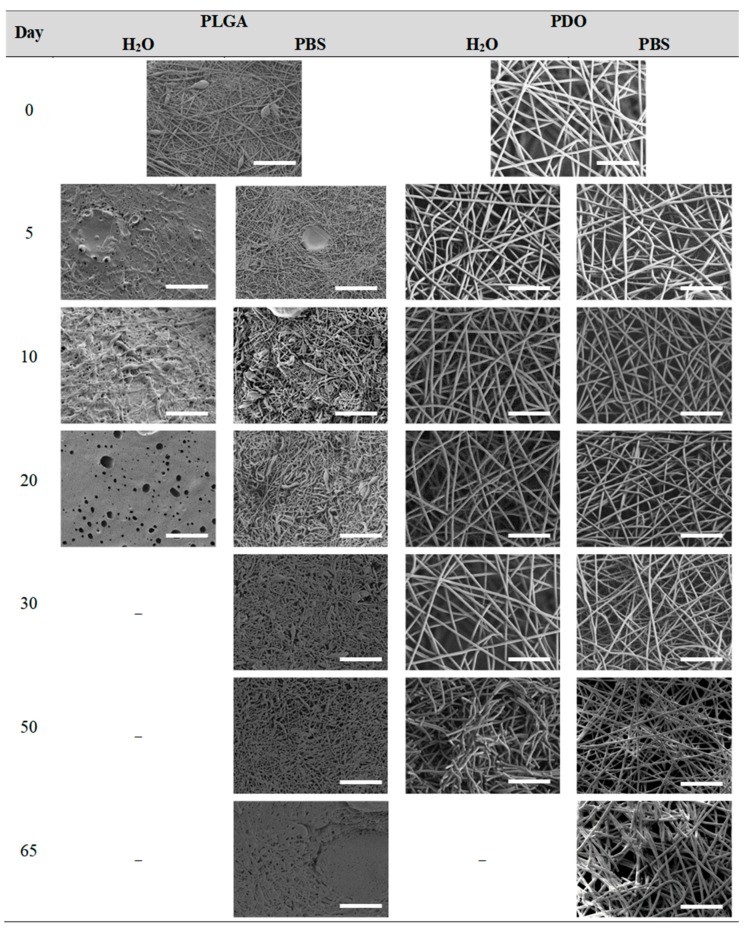
Surface morphology as a function of immersion time in ultra-pure water and PBS for PLGA and PDO scaffolds (500×: scale bar 50 µm).

**Figure 8:**
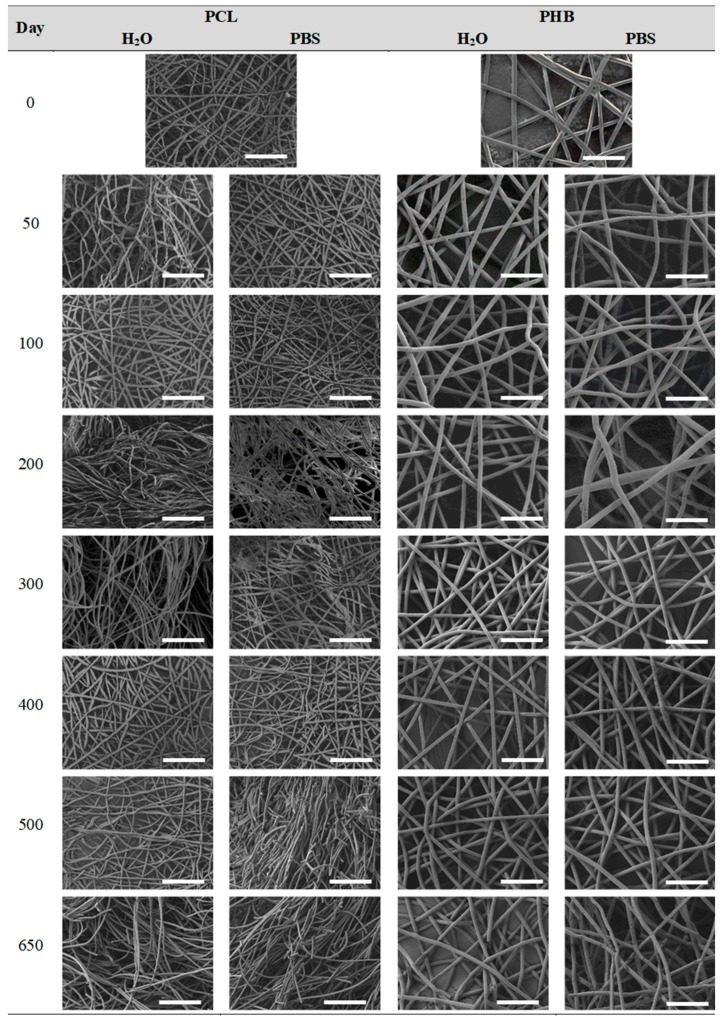
Surface morphology as a function of immersion time in ultra-pure water and PBS for PCL and PHB scaffolds (500×: scale bar 50 µm).

Finally, the change of the surface morphology of the scaffolds as a function of immersion time in ultra-pure water and PBS was monitored. The fibrous structure of the scaffold is required to remain for a certain period of time in order to allow cells to attach and proliferate [13]. The surface micrographs of PLGA and PDO are shown in Figure 7 while those of PCL and PHB scaffolds are shown in Figure 8. The average fibre diameters are gathered in Table 5.

Different behaviour was perceived in the fibrous morphology of the short-term degraded scaffolds. In the PLGA scaffolds, fibres swelled and coalesced along the immersion, as perceived in the fibre diameter increase [3]. As well, some microscopic pores appeared and grew in the surface of the fibres when immersed in water and, subsequently, the disintegration occurred around day 20. When immersed into PBS, the fibres also swelled, but the degradation process seemed to be slower than in water. Although swelling took place, conglutination was not completely reached in the scaffolds after 65 days of immersion. For PDO scaffolds, a slight reduction of the fibre diameter was found along the immersion in both media. The fibres did not coalesce and collapsed, probably due to the presence of the crystalline fraction. The degradation of the scaffolds occurred through fibre breakage in a longer timespan than for PLGA. The fibrous structure remained apparently unaffected until disintegration, which happened around day 60 of immersion.

In contrast, the fibrous structure of the PCL and PHB scaffolds remained almost unaffected during immersion, as revealed by the average fibre diameter [26]. Fibre breakage was perceived from day 500 onwards in the PCL scaffolds. After 650 days, the scaffolds were almost disintegrated as shown by the high degree of fibre rupture. Remarkable fibre breakage in PHB scaffolds was also found at 650 days of immersion. These observations confirm the high stability under hydrolytic conditions of these polymers that permitted to preserve the fibres unaltered until the last stages of immersion.

## 4. Discussion

Given the polyester nature of the scaffolds, the feasible ester bond scission and the generation of shorter polymer segments with new carboxyl groups and acidification of the media during immersion may take place [10]. In this line, due to the different polymers analysed, the changes in the molar mass are expected to depend on the chemical structure, but also on the scaffold crystalline morphology. As well, the variation of the fibrous structure of the scaffolds may be correlated to the above-cited behaviour. For a proper comparison, the overview of the degradation features of the different analysed scaffolds is gathered in Table 6.

The PLGA fibres swelled as a function of the immersion time, reducing the inter-fibre distance and tending to close the pores required for satisfactory cell adhesion and proliferation. The mainly amorphous morphology of this polymer may have contributed to rapid diffusion of the aqueous solvent into the fibres. Meanwhile, the hydrolytic degradation of the polymer molecules occurred from the very beginning of immersion, following a random scission pattern, as suggested by the increase in the polydispersity index. Consequently, the molar mass dramatically decreased and low molar mass compounds were released from the scaffold, promoting acidification of the surrounding media. Finally, the scaffold collapsed and released glycolic and lactic acid monomers and oligomers that were completely dissolved in the hydrolytic media.

The PDO scaffolds kept the fibrous morphology along the degradation until disintegration. The fibre diameter slightly diminished during immersion and small cracks perpendicular to the axis of the fibres were perceived. The presence of the ether bond in the backbone may have promoted the more progressive degradation behaviour of this material. The typical cold-crystallisation of this polymer disappeared along the immersion and an imperfect crystalline population was developed, which induced higher resistance than PLGA to coalesce and collapse. Nonetheless, the fibre erosion and breakage resulted in the progressive molar mass loss, mass-loss and the reduction of the pH due to the release of low molar mass species, as produced from the preferential bond scission of the ester linkages.

The morphology of the PCL fibres remained mainly unaffected along the immersion. The average fibre diameter slightly varied and the fibrous morphology persisted until disintegration. Crystallinity was developed in this scaffold due to the increase of mobility of short hydrolysed segments, aided by the plasticizing effect of water along with the temperature above the glass transition. As a consequence of having more crystalline domains with higher lamellar thickness, PCL was more capable of preventing the structure of its fibres from degradation. The molar mass followed an almost linear diminishing trend until complete disintegration. The polydispersity increased, suggesting a random chain scission phenomenon that resulted in dissimilar segment sizes during degradation. 

The hydrolytic behaviour of the PHB fibres revealed that the mass, the pH, the fibre diameter and the fibrous morphology remained constant until disintegration. Moreover, the crystallinity degree did not significantly varied during immersion. The highly stable crystalline structure of the PHB stimulated a slight and progressive chain scission process along the immersion, as suggested by the evolution of the molar mass distributions. The broad multi-modal behaviour of PHB remained almost unchanged as a function of the immersion time. However, the peak correlated to high molar mass segments decreased, while that of low molar mass polymer chains increased. This behaviour suggested preferential degradation of high molar mass domains. Overall, the PHB scaffolds were found to be the most stable of the evaluated materials, which revealed the slowest degradation behaviour when subjected to physiologic conditions.

## 5. Conclusions

The hydrolytic degradation patterns of scaffolds based on polyesters such as poly(lactide-co-glycolide) (PLGA), polydioxanone (PDO), polycaprolactone (PCL), and polyhydroxybutyrate (PHB) were monitored under in vitro simulated physiologic conditions. The PLGA and PDO scaffolds exhibited a short-term degradation performance, while those of PCL and PHB revealed a long-term and progressive degradation profile. Further evaluation of the behaviour of the scaffolds during immersion revealed that they possess different mechanisms of degradation, in which the decrease of the molar mass is strictly correlated to the polymer composition, but also to the scaffold crystalline structure, which will determine its subsequent biomedical application.

The results of this study may serve as a reference point in the design and selection of polyester-based electrospun scaffolds for biomedical applications, from the perspective of an adequate balance between the durability and degradation pattern under physiologic conditions.

## Figures and Tables

**Table 1:** Electrospinning conditions of the poly(lactide-co-glycolide) (PLGA), polydioxanone (PDO), polycaprolactone (PCL) and polyhydroxybutyrate (PHB) scaffolds.

Polymer (type)	Concentration (% wt)	Solvent (type)	Feed Rate (mL·h^−1^)	Voltage (kV)	Distance (cm)
PCL	18	DMF/CHCl_3_ 1:8	4	7.32/−5.54	25
PDO	8	HFIP	1	3.85/−3.35	21
PHB	23	CHCl_3_	1	7.80/−1.93	20
PLGA	30	DMF	1	9.15/−8.80	20

**Table 2:** Initial properties of the PLGA, PDO, PCL and PHB scaffolds.

Polymer	Constitutional Repetitive Unit (CRU)	Surface Morphology (500×)	Fibre Diameter Distribution	Average Diameter	ρ	ρ_s_
				(µm)	(g·cm^−3^)	(g·m^−2^)
**PLGA**	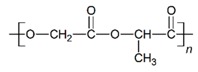	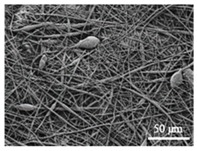	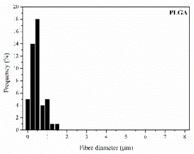	0.61	1.25	2.60
PCL	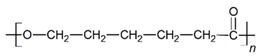	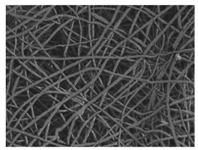	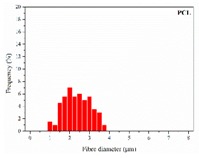	2.45	1.14	2.66
PDO	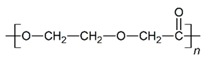	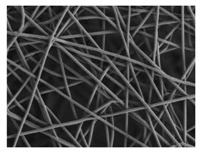	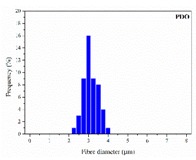	3.21	1.18	2.62
PHB	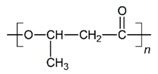	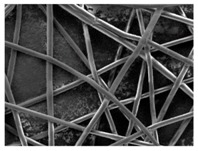	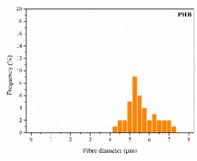	5.78	1.25	2.04

**Table 3:** Average molar mass in number as a function of the degradation time in ultra-pure water and PBS for PLGA, PDO, PCL and PHB scaffolds.

Immersion time (Days)	PLGA		PDO		Immersion Time (Days)	PCL		PHB	
	Mn (g·mol^−1^)					Mn (g·mol^−1^)			
	H_2_O	PBS	H_2_O	PBS		H_2_O	PBS	H_2_O	PBS
0	43,290		52,000		0	42,030		20,450	
5	36,080	38,210	30,800	50,450	50	36,080	34,500	18,490	19,510
10	26,020	25,780	28,080	48,880	100	30,010	28,820	15,700	17,040
20	12,690	15,850	16,620	30,260	200	27,810	25,390	14,900	14,850
30	-	8340	13,030	21,990	300	18,470	19,670	14,250	13,610
50	-	5960	-	14,460	400	8350	8380	13,650	12,350
65	-	4800	-	12,710	500	7520	5370	12,960	12,520
100	-	4360	-	9450	650	3340	3060	10,760	9440

**Table 4:** Peak temperature of the heat release during the glass transition (T_r-gt_) for the PLGA and the peak melting temperature (Tm) for the PDO, PCL and PHB scaffolds as a function of immersion time in ultra-pure water and PBS. Standard deviation between 0 and 1% was omitted for the sake of clarity.

Immersion Time (Days)	PLGA		PDO		Immersion Time (Days)	PCL		PHB	
	T_r-gt_ (°C)		T_m_ (°C)			T_m_ (°C)		T_m_ (°C)	
	H_2_O	PBS	H_2_O	PBS		H_2_O	PBS	H_2_O	PBS
0	54.5		107.9		0	63.0		170.9	
5	55.3	53.6	105.4	107.7	50	63.8	64.4	170.6	170.8
10	54.5	52.5	105.9	107.8	100	64.8	64.5	169.8	169.5
20	48.6	49.3	105.2	108.4	200	64.8	64.5	169.4	169.4
30	-	41.1	-	104.1	300	66.0	64.1	168.5	170.6
50	-	-	-	103.5	400	65.4	64.2	168.3	168.3
65	-	-	-	105.9	500	64.2	64.2	167.6	169.5
-	-	-	-	-	650	63.8	64.4	166.3	169.2

**Table 5:** Average fibre diameter as a function of the degradation time in ultra-pure water and PBS for PLGA, PDO, PCL and PHB scaffolds.

Immersion Time (Days)	PLGA		PDO		Immersion Time (Days)	PCL		PHB	
	Diameter (µm)					Diameter (µm)			
	H_2_O	PBS	H_2_O	PBS		H_2_O	PBS	H_2_O	PBS
0	0.610		3.214		0	2.458		5.783	
5	2.842	1.925	3.197	3.214	50	2.634	2.238	5.409	5.120
10	3.261	2.062	3.191	3.021	100	2.383	2.140	4.704	5.374
20	-	1.869	2.988	2.921	200	2.309	2.294	4.802	5.055
30	-	1.919	2.720	2.749	300	2.133	2.681	4.797	4.907
50	-	1.905	-	2.432	400	2.644	2.187	4.918	4.942
65	-	3.823	-	2.090	500	2.262	2.121	4.817	5.004
					650	2.150	2.455	4.755	4.861

**Table 6:** Overview of the hydrolytic degradation behaviour of PLGA, PDO, PCL and PHB when subjected to physiologic conditions (PBS, 37 °C).

	Poly(lactide-co-glycolide) (50:50) (PLGA)	Polydioxanone (PDO)	Polycaprolactone (PCL)	Polyhydroxybutyrate (PHB)
Origin	Synthetic	Synthetic	Synthetic	Natural
Microstructure	Amorphous	Semicrystalline	Semicrystalline	Semicrystalline
Temperature (37 °C)	Below the T_g_ (48 to 52 °C)	Above the T_g_ (−10 to −5 °C)	Above the T_g_ (−65 to −60 °C)	Above the T_g_ (0 to 5 °C)
Degradation pattern	Random chain scission, increase polydispersity	Preferential long-segment chain scission, reduce polydispersity	Random chain scission, increase polydispersity	Preferential long-segment chain scission, high polydispersity
Scaffold degradation mechanism (Adapted from [3])	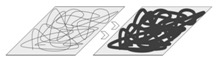	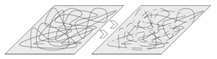	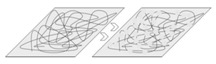	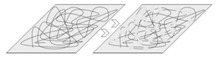
Hydrolytic degradation ultimately reaction	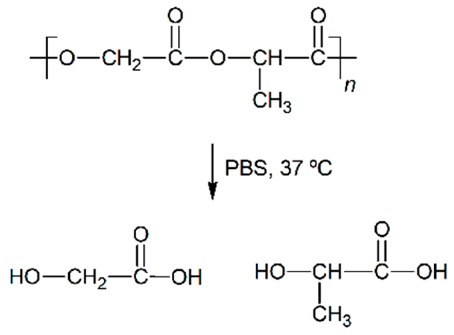	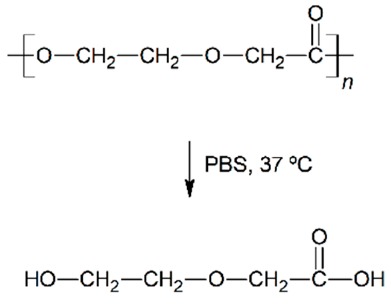	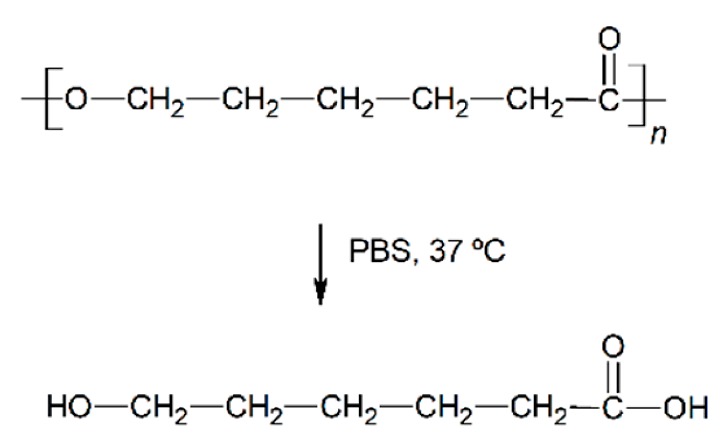	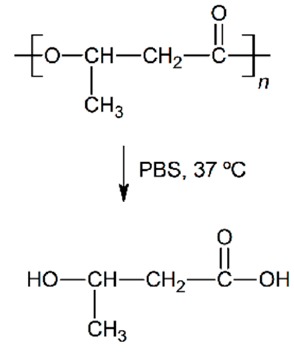
Degradation rate in physiologic conditions (50% M_n_ decrease)	15 days	30 days	300 days	650 days
Disintegration	125 days	125 days	650 days	650 days
pH reduction (<7)	65 days	50 days	No change	No change
Changes in the microstructure	Disappearance of structural relaxation and separation of glass transitions of the co-monomers (PLA, PGA)	Crystalline development of lower lamellar thickness first, and then perfection of these structures	Crystalline development of higher lamellar thickness domains	High stability of crystalline domains with a slight decrease of lamellar thickness
